# Early-onset basal cell carcinoma; wide case series at a single tertiary center in middle Anatolia

**DOI:** 10.14744/nci.2020.78872

**Published:** 2022-07-08

**Authors:** Ayse Nur Ugur Kilinc, Zeynep Bayramoglu, Yasar Unlu

**Affiliations:** Department of Pathology, Konya Training and Research Hospital, Konya, Turkiye

**Keywords:** Basal cell carcinoma, early-onset, young patients

## Abstract

**OBJECTIVE:**

Although basal cell carcinoma (BCC) is prevalent in the older population, it can be rarely seen in younger people. This study aims to investigate the risk factors and characteristics of BCC in young patients.

**METHODS:**

Pathology reports in a single tertiary care institution between 2010 and 2020 were retrospectively reviewed. Patients under the age of 35 who were diagnosed with BCC were included for the analysis. Data were gathered from medical records and pathology reports. Demographic characteristics, accompanying pathologies, and clinical findings of these patients were analyzed.

**RESULTS:**

There were a total of 32 patients in our cohort. Out of 32 patients, 20 were female and 12 were male. One male patient and five female patients were younger than 18. An accompanying risk factor (Gorlin syndrome, XP, renal transplantation, etc.) was present in six patients. There were no known additional diseases or risk factors in 26 patients. Metastasis and recurrence were not reported in any of our patients. Contrary to the information in the literature, the patients with BCC at a young age were not found more aggressive in our study.

**CONCLUSION:**

Contrary to the information in the literature, the patients with BCC at a young age were not found more aggressive in our study. Understanding the risk factors associated with BCC is essential for designing prevention strategies and favoring early diagnosis. Awareness of early-onset BCC aids in early diagnosis and treatment of the disease. Therefore, BCC should be in the differential diagnosis of skin lesions in the young population. In addition, when we encounter early-onset BCC, we should not forget the accompanying risk factors and syndromes.

## Highlight key points

- Since BCC is not very rare in the young population, BCC should be in the differential diagnosis of skin lesions in the young population.
- Contrary to what is known, early onset BCC may not be more aggressive.
- Risk factors and BCC-related syndromes should be kept in mind when encountered with early-onset BCC.

Basal cell carcinoma (BCC) is a carcinoma derived from basal cells of the interfollicular epidermis and/or hair follicle. BCC’s exhibit morphological variation, but they invariably contain islands or nests of peripherally palisaded basaloid cells with hyperchromatic nuclei and scant cytoplasm [1, 2].

BCC is the most common malignancy in humans. Incidence rates are inversely related to geographical latitude and higher in the light-skinned population. The risk of BCC steadily increases by age and sun exposure. In the last classification of WHO, skin tumors report that 64% of BCCs are seen in the head and neck 24% and are seen in the trunk in adults [1].

However, the incidence of BCC is more common in the elderly it is strikingly increasing in younger age groups, particularly among women [3]. It is reported in the literature that there may be various risk factors in the formation of early-onset BCC. Exposure to indoor tanning and sun exposure is shown as the main responsible for risk factors [4]. Early-onset BCC has been associated with various syndromes and diseases in the literature. Childhood-onset of BCC has previously been associated with albinism, Bazex syndrome, basal cell nevus syndrome, nevus sebaceous, solid organ transplants, xeroderma pigmentosum, and radiotherapy-treated cancer [2, 5].

BCCs, except for some rare subtypes, have a good prognosis and do not show metastasis or recurrence. In the WHO classification of skin tumors, BCC subtypes are classified as high risk (basosquamous, sclerosing, infiltrating, sarcomatoid differentiation, and micronodular) and low risk (nodular, superficial, pigmented, infundibulocystic, and fibroepithelial) BCC subtypes [1]. This study aims to investigate the risk factors and characteristics of BCC in young patients.

## MATERIALS AND METHODS

After obtaining the approval of the local ethics committee of SBU Konya Training and Research Hospital TUEK (date: 02/07/2020, decision no: 40/10). Pathology reports in a single tertiary care institution between 2010 and 2020 were retrospectively reviewed. All patients who were diagnosed with BCC were detected. Demographic data were gathered from medical records and pathology reports. Patients under the age of 35 who were diagnosed with BCC were examined in detail. Age, sex, comorbidities, risk factors for BCC, and pathological characteristics were analyzed patients under thirty-five. Pathology reports and slides were reviewed. Representative pictures were included in the study. The study was conducted in compliance with the principles included the Declaration of Helsinki.

### Statistical Analysis

Statistical analysis of all data was performed using SPSS (Statistical Packages for the Social Sciences, software, edition 21, SPSS Inc. Chicago, USA).

## RESULTS

A total of 1717 patients were diagnosed with BCC at our hospital between 2010 and 2020. The mean age was 70.6±13.8 (Table 1). Out of 1717, 32 patients (1.86%) were under the age of 35 at the time of diagnosis. Twenty patients were female and 12 were male. The mean age was 26.3±9.4 (range 6–35). One of the male patients (age 14) and 5 of the female patients (range 6–12) were younger than 18 (Table 1).

**Table 1:** Demographic data of patients with basal cell carcinoma

	Total number	Age at first diagnosis, y Mean±SD
All patients with BCC	1717	70.6±13.8
Female	859	70±14.2
Male	858	76.5±13.3
Patients under <35 with BCC	32	26.3±9.4
Female	19	24.6±10.3
Male	13	28.5±7.5
Patients under <18 with BCC	6	10.8±2.4
Female	5	10.2±2.2
Male	1	14±0

BCC: Basal cell carcinoma.

Patients were equally distributed between the first and second half of the study period. No significant increase in the diagnosis of BCC in the younger population was noted in recent ten years. The histological subtypes were nodular (29 patients), superficial spreading (two patients), and pigmented BCC (1 patient). Low-risk subtypes were observed in all of our cases and none of our patients had high-risk subtypes (Table 2).

**Table 2:** Histological subtypes, tumor site, and focality of patients with basal cell carcinoma under age 35

Histological subtypes	
Nodular	29/32
Superficial-spreading	2/32
Pigmented	1/32
Tumor site	
Head and neck	31/32
Upper limb	1/32
Trunk-lower limb	0
Tumor focality	
Unifocal	29/32
Multifocal	3/32

BCC: Basal cell carcinoma.

The location of BCC was head in all of our patients, except one patient had BCC in the upper extremity. Three patients (percentage 9%) had multifocal lesions, while others had a single lesion. One of the patients with multifocal lesions had a history of xeroderma pigmentosum. The other two patients also demonstrated BCC lesions on the face but they had no risk factors.

An accompanying risk factor was present in six patients (18%). The risk factors were Gorlin-Goltz syndrome, renal transplant history, Sebaceous nevus, Xeroderma pigmentosum, Epidermolysis bullosa, and extended sun exposure (living in Africa for 9 years) (Table 3). Additional comorbidities were detected in the other seven patients (Table 4). There were no known additional diseases or risk factors in the remaining 18 patients 56.2% (18/32). Metastasis and recurrence were not reported in any of our patients. None of our patients have artificial ultraviolet (UV) radiation, UV beds, or an indoor tanning history.

**Table 3:** Early-onset basal cell carcinoma with presence of known risk factors for basal cell carcinoma

Risk factors	Age	Gender	Localization	Histological subtype
Gorlin-Goltz syndrome	30	M	Scalp midline	Superficial
Renal transplant history	30	M	Left malar region	Nodular
BCC developing from Sebaceous nevus	21	M	Scalp	Nodular
Xeroderma pigmentosum	17	F	Forehead, cheek, infraorbital area, and left intranasal area (multiple lesions)	Nodular
Epidermolysis bullosa	6	F	?	Nodular
Personal history of living in Africa for nine years	25	M	Medial left orbit	Nodular

BCC: Basal cell carcinoma.

**Table 4:** Early-onset basal cell carcinoma with comorbidities

Comorbidity	Age	Gender	Localization	Histological subtype
Polycystic ovary syndrome	25	F	Scalp	Nodular
Breast fibroadenoma	26	F	Nasal lateral	Nodular
Antipsychotic drug use	30	M	Scalp	Nodular
Irritable bowel disease	32	M	Arm	Superficial
Diabetes mellitus (type II)	35	M	Two lesions Nasolabial region, medial of eye	Nodular
Polymorphous light eruption	32	F	Right cantal region	Nodular Nodular
Xerosis cutis	21	F	Frontal region	

## DISCUSSION

There are various case reports and reviews in the literature regarding early-onset BCCs, and a few single-center case series have been reported [3, 5–18]. This study represents one of the biggest series of BCC in young patients in a single tertiary institution.

In our series, BCC cases under the age of 35 constitute 1.8% of all BCC cases, while this rate was found to be 2.5% in another study with a similar number of samples in Spain [10]. This result shows us that even if the incidence of BCC increases at a young age, it still constitutes a small part of all BCC cases. The low incidence of BCC in young patients could cause diagnostic delays due to a low index of suspicion. When we look at the gender distribution of our patients in all patients with BCC, interestingly, we see that there are equal numbers of male and female patients. In our patients under 35 and under 18, the female to male ratio was 1.46 (19/13) and 5 (5/1), respectively. It is striking that the average age of all age groups and our patients under 35 years old is slightly higher in men compared to women.

We know that BCC is primarily seen in the sun-exposed areas. As in other studies in the literature, the location of the head and neck stands out in our early-onset BCC cases [5, 10, 17]. Childhood-onset of BCC has previously been associated with albinism, Bazex syndrome, BCC nevus syndrome, nevus sebaceous, solid organ transplants, xeroderma pigmentosum, and radiotherapy-treated cancer [4, 12, 19, 20]. Similarly, some of these risk factors (Gorlin, XP, renal transplant, etc.) were present in six out of 32 patients. Interestingly, xerosis cutis and the polymorphic light eruption, which are the skin diseases, also, were not previously shown to be associated with BCC.

When we look at pediatric patients (0–18), there were known risk factors in two patients, while no known risk factors were found in four patients. Pathogenesis in children without risk factors is not fully known, and specific genetic and iatrogenic factors have not been defined yet. These patients may have mosaic genetic conditions [21].

**Figure 1:**
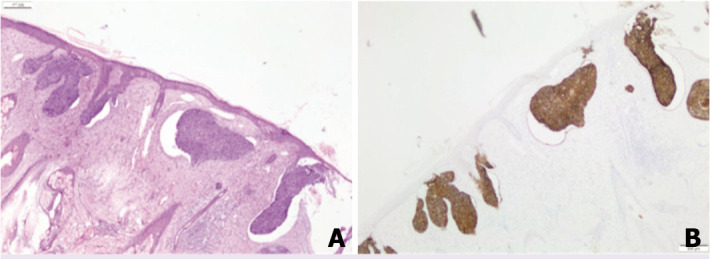
Superficial spreading basal cell carcinoma, 31-year-old male with Gorlin-Goltz syndrome ×40 H&E Stain **(A)**, ×40 Ber-ep 4 Stain **(B)**.

**Figure 2:**
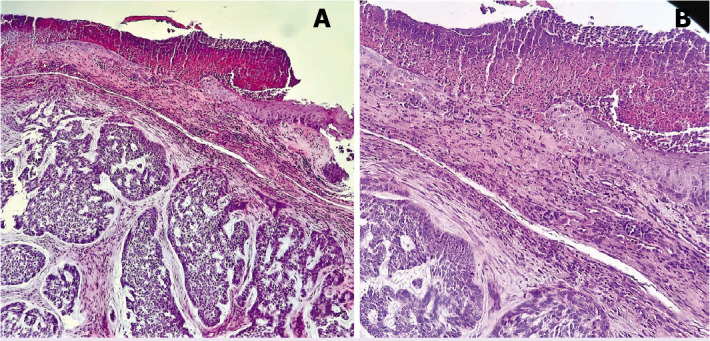
Nodular basal cell carcinoma, 6-year-old female with epidermolysis bullosa ×40 H&E Stain **(A)**, ×100 H&E Stain **(B)**.

In the literature, BCC in young patients independent of a syndrome or risk factor (idiopathic BCC) is thought to be more aggressive [9, 11, 12]. We found that 82% of our cases had idiopathic BCC. However, our results do not support this information. In our series, all subtypes were low-risk histological subtypes (Fig. 1, 2). Recurrence and aggressive subtypes were not observed in any of the cases with BCC at an early age in our series. The limitation of our study is that we could not add any molecular analysis for early-onset BCC.

## Conclusion

Contrary to the information in the literature, the patients with BCC at a young age were not found more aggressive in our study. Understanding the risk factors associated with BCC is essential for designing prevention strategies and favoring early diagnosis. Awareness of early-onset BCC aids in early diagnosis and treatment of the disease. Therefore, BCC should be in the differential diagnosis of skin lesions in the young population. Furthermore, when we encounter early-onset BCC, we should not forget the accompanying risk factors and syndromes.
